# No Difference between Spinal Anesthesia with Hyperbaric Ropivacaine and Intravenous Dexmedetomidine Sedation with and without Intrathecal Fentanyl: A Randomized Noninferiority Trial

**DOI:** 10.1155/2022/3395783

**Published:** 2022-01-13

**Authors:** Seung Cheol Lee, Tae Hyung Kim, So Ron Choi, Sang Yoong Park

**Affiliations:** Department of Anesthesiology and Pain Medicine, Dong-A University College of Medicine, Busan, Republic of Korea

## Abstract

To enhance the duration of single-shot spinal anesthesia, intrathecal fentanyl and intravenous dexmedetomidine are widely used as adjuvants to local anesthetics. This noninferiority trial evaluated whether hyperbaric ropivacaine alone can produce a noninferior duration of sensory block in comparison to hyperbaric ropivacaine with intrathecal fentanyl in patients under dexmedetomidine sedation. *Methods*. Fifty patients scheduled for elective lower limb surgery under spinal anesthesia were randomly assigned in a double-blind fashion to receive either hyperbaric ropivacaine 15 mg (Group R) or hyperbaric ropivacaine 15 mg with intrathecal fentanyl 20 *μ*g (Group RF). Intravenous dexmedetomidine (1 *μ*g/kg for 10 min, followed by 0.5 *μ*g/kg/h) was administered in both groups. The primary outcome of this study was the time to two-dermatomal regression of sensory block. The noninferiority margin for the mean difference was −10 min. Characteristics of the block, intraoperative and postoperative side effects, postoperative pain score, and analgesic consumption were assessed as secondary outcomes. *Results*. There was no difference in the two-dermatomal regressions of sensory block between the two groups (Group *R* 70.4 ± 10.2 min, Group RF 71.2 ± 12.4 min, *p* = 0.804) with a mean difference of 0.8 min (−7.2 to 5.6, 95% confidence interval). Thus, the noninferiority of hyperbaric ropivacaine alone was established. There were no significant differences in the secondary outcomes between the two groups. *Conclusions*. Under intravenous dexmedetomidine sedation, the duration of spinal anesthesia with hyperbaric ropivacaine alone was noninferior to that of hyperbaric ropivacaine with intrathecal fentanyl. This suggests that addition of intrathecal fentanyl to hyperbaric ropivacaine may not be necessary in patients receiving intravenous dexmedetomidine.

## 1. Introduction

Spinal anesthesia is a common technique that provides fast and deep anesthesia in lower limb surgeries. However, it is performed in a single injection and the duration of anesthesia cannot be prolonged during surgery if required [1]. In order to compensate for this problem, several adjuvants are widely used with local anesthetics [2–4]. For example, using opioids in local anesthetics can prolong the duration of block as opioid receptors in the spinal cord are isolated [2]. Among these, intrathecal fentanyl is the most commonly used anesthetic adjuvant, and its effect has been proven in previous studies [2, 5, 6]. However, adverse effects, such as respiratory depression, nausea and vomiting, pruritus, and urinary retention, have been reported with intrathecal fentanyl use.

Ropivacaine, an amide local anesthetic, has a chemical structure similar to that of bupivacaine, which is widely used as a spinal anesthetic [7, 8]. It is relatively less potent than bupivacaine and has a higher therapeutic index and better safety profile. The main advantage of ropivacaine is its lower toxicity, especially lower cardiotoxicity, after accidental intravascular injection [8, 9]. Hyperbaric ropivacaine prepared by adding glucose to ropivacaine has been used for spinal anesthesia in lower limb surgeries [7, 10]. It has faster onset, a greater success rate of analgesia, and faster recovery of the sensory block than with plain ropivacaine [10]. Compared with bupivacaine, ropivacaine has shorter duration of anesthesia, but it is sufficient for lower limb operations along with a thigh tourniquet; moreover, its shorter duration of effect has been identified as a potential benefit [6, 10, 11]. However, because of its short duration of action, some adjuvants are often required to achieve satisfactory anesthesia [6, 12]. Therefore, addition of intrathecal fentanyl to hyperbaric ropivacaine can prolong the duration of anesthesia [6, 13].

Dexmedetomidine, an *α*-2 adrenergic agonistic agent, has been widely used as an adjuvant to spinal anesthesia intravenously or intrathecally and offers satisfactory sedation if used intravenously, while reducing the risk of respiratory depression [12, 14, 15]. Its effects have been associated with postoperative analgesia and a decrease in the first 24-hour opioid use following surgery [15, 16]. However, the use of intravenous dexmedetomidine may be associated with an increased risk of bradycardia and hypotension [12, 15, 17].

Intrathecal opioids, especially fentanyl, are commonly used even with intravenous dexmedetomidine sedation, but it is unclear whether they can produce additional benefits in comparison with hyperbaric ropivacaine alone. Intrathecal fentanyl has a prolonged duration of action but also has adverse effects [2, 5]. It is not necessary to administer intrathecal fentanyl if intravenous dexmedetomidine alone could prolong the duration of block sufficiently for lower limb surgeries. Currently, intrathecal fentanyl is still commonly used during spinal anesthesia, even under intravenous dexmedetomidine sedation. Thus, this study was designed to evaluate the effects of intrathecal fentanyl by comparing ropivacaine plus fentanyl solution with ropivacaine alone in patients under dexmedetomidine sedation. We also investigated the intraoperative adverse effects and postoperative analgesic profiles.

## 2. Materials and Methods

This prospective study was a randomized, double-blind, noninferiority trial conducted at a single center. It was approved by the Institutional Review Board of Dong-A University Hospital (DAUHIRB-20-085) and was registered with CRIS.NIH.go.kr (identifier KCT0005116; date of registration: June 12, 2020).

Patients aged 20–70 years with ASA I/II undergoing scheduled elective lower limb surgery under spinal anesthesia were recruited and randomized using computer-programmed randomization between July 2020 and March 2021. All patients provided written informed consent for the study. Patients with contraindications to spinal anesthesia, including patient refusal and those with local infection at the puncture site, coagulopathy, and allergy to the drugs administered in this study, were excluded. Patients with previous spinal surgery, spinal deformities, severe spinal stenosis, morbid obesity with body mass index >30 kg/m^2^, height under 150 cm or over 180 cm, and cognitive impairment, including those who were unable to communicate and cooperate, were excluded. Patients with severe cardiovascular impairment, such as valve diseases, heart block, and left ventricular dysfunction, were also excluded.

Patients were randomly assigned to the hyperbaric ropivacaine group (Group R) or hyperbaric ropivacaine with adjuvant fentanyl group (Group RF). Patient allocation was only open to the anesthesiologist who prepared the anesthetics and performed spinal anesthesia. After the procedure, the anesthesiologist was excluded from the study, and the outcome assessor continued the study and investigated the parameters. Thus, the outcome assessors and participants were blinded to group allocation. A single orthopedic surgeon who was also blinded to the group allocation performed the operations for every patient.

In both groups, we reviewed the medical records to evaluate the patients' physical data, medical history, and functions. All patients fasted overnight, and intravenous access was secured. Patients were under standard monitoring after entering the operating room and underwent spinal anesthesia at the L4/5 interspace with a 25G Quincke bevel spinal needle (Uniever, Saitama, Japan) in the lateral decubitus position. In Group *R*, the patients received intrathecal hyperbaric ropivacaine 15 mg alone. In Group RF, hyperbaric ropivacaine 15 mg combined with fentanyl (Hana Pharmaceutical, Seoul, Korea) 20 *μ*g was administered. The study solution, hyperbaric ropivacaine, was prepared with 2 mL of 0.75% ropivacaine (Hanlim Pharmaceutical, Seoul, Korea) and 1 mL of 20% dextrose (JW Pharmaceutical, Seoul, Korea) using a strict aseptic technique [11, 18]. The total volume of anesthesia was 3 mL in Group *R* and 3.4 mL in Group RF.

All spinal anesthesia procedures were performed by a single experienced anesthesiologist. After intrathecal injection, patients were placed in the supine position, followed by administration of oxygen at 5 L/min. A separate investigator, who was blinded to the study solutions, assessed the extent of sensory and motor blocks. The assessments of sensory block by pinpricking (at midclavicular line using a short beveled 26-gauge needle) were performed at 1, 5, 10, 15, and 20 min after intrathecal injection and then every 10 min until the patients were discharged from the recovery room. Surgery was performed after the sensory block of the T10 dermatome was established.

Motor block assessments were performed immediately after sensory block assessments using a modified Bromage scale (0 = no paralysis, 1 = unable to raise the extended leg, 2 = unable to flex the knee, and 3 = unable to flex the ankle). During surgery, the motor block could not be assessed and was started again immediately after surgery. Motor assessments were continued until the patient was discharged from the recovery room.

Dexmedetomidine (Kyungbo Pharmaceutical, Seoul, Korea) was prepared with a dilution of 4 *μ*g/mL with saline and administered intravenously 20 min after spinal anesthesia. Both groups received 1 *μ*g/kg of dexmedetomidine for 10 min, followed by 0.5 *μ*g/kg/h. After administration, the bispectral index (BIS) monitoring was also established. The level of sedation 20 min after the administration of dexmedetomidine was evaluated using the BIS. The administration of dexmedetomidine was stopped when the surgeon started placing the skin sutures. An intravenous patient-controlled analgesia (PCA) pump (Ace Medical, Korea) was connected at the end of the surgery. The 100 mL PCA bag contained nefopam 0.5 mg/mL, fentanyl 15 *µ*g/mL, and ramosetron 0.6 mg. It was set to release 0.8 mL bolus, and the lockout time was 10 min. Baseline infusion was administered at 0.8 mL/h.

During anesthesia, noninvasive blood pressure, electrocardiogram, heart rate (HR), and oxygen saturation were monitored and recorded at 5 min intervals. Hypotension was defined as a 30% or greater decrease in systolic pressure from baseline, and bradycardia was defined as HR of <50 beats per minute. The patient with hypotension was treated with intravenous ephedrine at bolus doses of 5–10 mg and fluid replacement. The patient with bradycardia was treated with intravenous atropine 0.5 mg. Complications, such as hypotension, bradycardia, nausea, vomiting, pruritus, shivering, and dyspnea were also recorded.

Patients were carefully observed in the recovery room after surgery and then discharged to their wards. In the recovery room, the same investigator assessed the extent of sensory and motor blocks. Patients were educated to record their pain and complications on a sheet, which was returned 24 hours after the surgery. In addition, patients were informed to request for rescue analgesics if the pain was scored ≥4 on the numerical rating scale (NRS) on postoperative day 0. Intravenous pethidine 25 mg and/or oral tramadol 75 mg were administered as rescue analgesics if required. The cumulative dose of rescue analgesics and total analgesics, including PCA pump, were recorded 24 hours after surgery. Additionally, the time to the first feeling of pain, time to the first request of analgesics, and time to the first micturition were measured. Possible side effects, including nausea, vomiting, and pruritus, were recorded.

### 2.1. Statistical Analysis

The primary outcome measured was the time to two-dermatomal segment regression of sensory blockade below the maximum level. This study aimed to assess the noninferiority of the time to two-dermatomal segment regression of sensory blockade in patients who received ropivacaine compared with that of those who received ropivacaine and fentanyl. Considering surgical time in lower limb surgery, the noninferiority margin for the mean difference of the primary outcome was predetermined −10 min [19, 20]. The independent *t-*test was employed to test the noninferiority of the primary outcome (the null hypothesis was that the mean difference in the two-dermatomal segment regression time was greater than −10 min. The alternative hypothesis was that the mean difference in the time to two-dermatomal segment regression was less than −10 min). The sample size was determined by assuming a two-dermatomal segment regression time of 139 min with a standard deviation (SD) of 13.8 min [21]. We needed 25 participants in each group for a power of 0.8 and an alpha error of 0.05. Considering a dropout rate of 10%, a sample size of 28 patients for each group was chosen.

The data are presented as frequency and percentage for the categorical variables and mean ± SD/median (interquartile range (IQR)) for the numeric variables. The differences in the characteristics of the study participants were compared across the subgroups with chi-square test or Fisher's exact test for the categorical variables and independent *t-*test or Mann–Whitney's *U* test for the continuous variables as appropriate. We performed the Shapiro–Wilk test to check if the data distribution was normal. All statistical analyses were conducted using SPSS 26.0, and a *p* value of <0.05 was considered statistically significant.

## 3. Results

**Figure 1:**
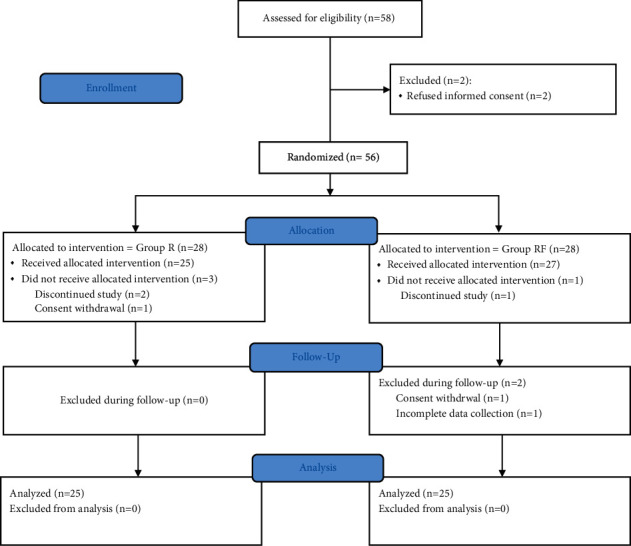
Flowchart of the study.

**Figure 2:**
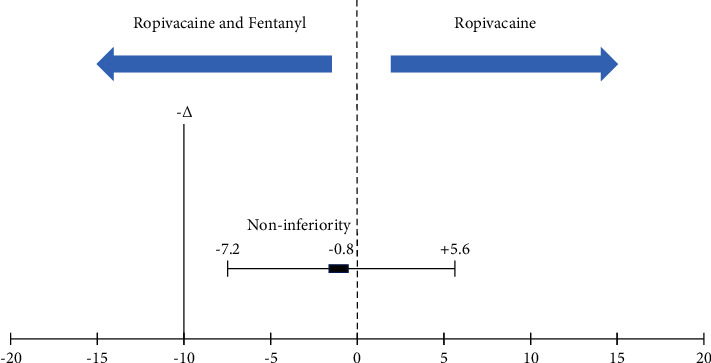
Noninferiority diagram with the difference between Group *R* and Group RF in the time for two-segment regression of sensory block.

Fifty-eight patients were assessed for eligibility, and 56 patients were randomized. Six patients were excluded due to various reasons (Figure 1). Thus, 50 patients completed the study, and the groups were comparable with regard to the baseline characteristics (Table 1). The primary outcome, that is, the time to two-dermatomal regression of sensory block, was 70.4 ± 10.2 min for Group *R* and 71.2 ± 12.4 min for Group RF (Table 2). The mean difference in the time for two-dermatomal segment regression of sensory blockade was 0.8 min (95% confidence interval, −7.2 to 5.6; *p* = −0.8 for noninferiority). The lower limit of the 95% confidence interval for the mean difference was −7.2 min; thus, the predetermined criteria for noninferiority were met between Group *R* and Group RF (Figure 2).

The other characteristics of anesthesia are summarized in Table 2. The maximum block between Group *R* and Group RF was not significantly different. The BIS at 20 min after intravenous dexmedetomidine administration did not differ between the two groups. There were no significant differences between the two groups in terms of block characteristics, including onset time and recovery time. No significant differences were found between the two groups in terms of the time to the first micturition (Table 2), intraoperative side effects, and postoperative side effects (Table 3). The postoperative pain score and cumulative dose of analgesics in 24 hours after surgery had no difference between the two groups; however, the mean time to the first request of analgesics in Group *R* (176.8 ± 90.4 min) was shorter than that in Group RF (252.0 ± 190.9 min), but not statistically significant (Table 4).

## 4. Discussion

The purpose of this study was to evaluate the additional effects of fentanyl as an adjuvant in spinal anesthesia with hyperbaric ropivacaine under dexmedetomidine sedation. This study was designed as a noninferiority trial to demonstrate that hyperbaric ropivacaine alone can offer sufficient duration and quality of block in comparison with hyperbaric ropivacaine with additional fentanyl in patients under sedation with intravenous dexmedetomidine. The results imply that intrathecal fentanyl may not be necessary in spinal anesthesia with ropivacaine for lower limb surgery if intravenous dexmedetomidine is administered. In addition to block characteristics, there were also no significant differences in postoperative pain score, requirement of analgesics, and adverse effects associated with the block between the two groups.

Considering the short duration of ropivacaine, we enrolled patients who were scheduled to undergo elective lower limb surgery. Whiteside et al. and Dar et al. conducted studies to compare the effects of ropivacaine with bupivacaine for spinal anesthesia [7, 11]. In their studies, 15 mg of hyperbaric ropivacaine alone could provide sufficient anesthesia for lower limb surgery and hip surgery. Since our study was designed to administer intravenous dexmedetomidine in both groups, we considered that 15 mg of hyperbaric ropivacaine is safe for lower limb surgery. Glucose-containing hyperbaric ropivacaine can produce predictable and a reliable block for a wide range of surgeries in comparison with isobaric ropivacaine [10, 22]. However, hyperbaric ropivacaine has a relatively short recovery profile and is commercially unavailable; thus, it should be prepared immediately before injection [7]. Despite these disadvantages, the advantages of more rapid regression of sensory and motor blocks, earlier mobilization, and shorter time to the first micturition could be preferable in an ambulatory setting [7, 23]. As ambulatory surgery has recently become the mainstream approach, such a profile may be beneficial. Although ropivacaine for spinal anesthesia has many advantages, the relatively short duration of its action during prolonged surgery could be a serious problem. Thus, during spinal anesthesia with ropivacaine, anesthesiologists should carefully determine which adjuvants should be administered. Various intrathecal adjuvants, such as opioids and *α*-2 agonists (clonidine and dexmedetomidine), can be administered along with ropivacaine [24, 25]. In a previous study, intrathecal dexmedetomidine demonstrated prolonged duration of sensory block with minimal side effects [24, 26]. It can also be administered intravenously for sedation during spinal anesthesia and has gained popularity due to the prolonged action of the block and patient satisfaction [12, 14]. In addition, unlike intrathecal administration, intravenous administration of dexmedetomidine can be controlled simultaneously by the anesthesiologist if required. Thus, our study aimed to determine the role of intrathecal adjuvants during intravenous dexmedetomidine sedation. Among them, fentanyl is one of the most commonly used adjuvants, and previous studies with ropivacaine have demonstrated the facilitatory effects of intrathecal fentanyl [5, 6, 13]. However, intrathecal fentanyl is associated with adverse effects, such as pruritus, nausea, and vomiting. Hence, its benefits and risks need to be reviewed in case of dexmedetomidine sedation because dexmedetomidine itself can prolong the duration of the block. Our study outcomes demonstrated that spinal anesthesia with ropivacaine alone was a noninferior block compared with ropivacaine with intrathecal fentanyl adjuvant under intravenous dexmedetomidine infusion.

The absence of intrathecal fentanyl can offer several advantages, in addition to unnecessary opioid use and medical costs. The potential risks of infection and neurotoxicity can arise in the administration of adjuvants [19, 27]. Moreover, fentanyl is used off-label since only morphine and baclofen are approved for intrathecal administration by the Food and Drug Administration [27]. In our study, adverse effects associated with the use of intrathecal fentanyl were not significantly different from those observed in patients without additional fentanyl administration. In actual clinical practice, significant side effects are rarely encountered with the usual 20 *μ*g intrathecal dose, but anesthesiologists should be aware about the possible adverse effects [19]. Postoperative pain and consumption of analgesics were not significantly different between the two groups, but the duration to request for the first analgesics in Group *R* was 75.2 min shorter than in that in Group RF, which while not statistically significant, could be clinically meaningful. Park et al. conducted a study to evaluate the effects of intrathecal fentanyl 20 *μ*g in spinal anesthesia with bupivacaine during dexmedetomidine sedation [19]. Their study also did not report any significant differences in the adverse effects after fentanyl administration than without additional fentanyl.

Intravenous dexmedetomidine administration offers several advantages, such as sedation without respiratory depression, postoperative analgesia, and a decrease in the first 24-hour opioid use after surgery [12, 15, 16]. However, the risk of bradycardia is increased. In our results, there were no significant differences in the BIS at 20 min after infusion, postoperative pain score, and cumulative dose of opioids in case of additional intrathecal fentanyl. In both groups, intraoperative bradycardia was observed at a high rate, and hypotension was observed in one patient; however, the difference was not significant. According to our results, the limiting usefulness of dexmedetomidine is the concern regarding peripheral *α*2-receptor stimulation resulting in hypotension and bradycardia [28]. Therefore, anesthesiologists should be prepared for bradycardia when administering intravenous dexmedetomidine.

Our study has some limitations. Because saline was not used as a control, the doses between the two groups were different. Because we wanted to observe the actual efficacy of ropivacaine alone without adjuvant fentanyl, our study did not intentionally eliminate the difference in volume of 0.4 mL between the two solutions. Different volumes of study solutions could affect the block characteristics. The attempt at blinding could have been improved if the same volume of the two solutions was used. Further, as this study did not have a control group without dexmedetomidine use, a third arm of the study could aid in evaluating the block characteristics and side effect profile of dexmedetomidine.

## 5. Conclusions

This study demonstrated that the duration and quality of block with hyperbaric ropivacaine alone is noninferior to that with hyperbaric ropivacaine with fentanyl under dexmedetomidine sedation during lower limb surgery. Intraoperative and postoperative side effects and analgesic profiles were not significantly different between the two groups. Based on this result, routine intrathecal fentanyl administration may be worth considering and should be used according to the indications during intravenous dexmedetomidine sedation. Further studies are required to evaluate other intrathecal adjuvants under dexmedetomidine sedation.

## Figures and Tables

**Table 1:** Patient characteristics and surgical data.

	Ropivacaine (*n* = 25)	Ropivacaine-fentanyl (*n* = 25)	*p* value
Sex, F/M	8/17	11/14	0.561
Age, years	46.2 (14.7)	44.8 (16.2)	0.750
Weight, kg	72.2 (9.9)	71.9 (14.6)	0.934
Height, cm	166.9 (9.0)	166.0 (9.0)	0.729
Body mass index, kg/m^2^	25.9 (2.4)	25.9 (3.5)	1.000
ASA I/II	9/16	8/17	1.000
Operation time, min	85.6 (34.9)	81.80 (36.7)	0.709
Anesthesia time, min	112.6 (37.2)	106.4 (38.7)	0.566

*Type of surgery*			
Lower leg	5 (20)	5 (20)	0.788
Ankle	15 (60)	13 (52)	
Foot	5 (20)	7 (28)	
Number of patients treated with atropine	14 (56)	15 (60)	0.774
Number of patients treated with ephedrine	1 (4)	1 (4)	1.000

Data are presented as mean (SD) or number (%) of patients. The *p* values are the results of the Mann–Whitney *U* test for the continuous variables and the *χ*^2^ test or Fisher's exact test for the incidence variables between the groups.

**Table 2:** Characteristics of the block.

	Ropivacaine (*n* = 25)	Ropivacaine-fentanyl (*n* = 25)	*p* value	Difference (95% CI)
Maximum block	T6 (T4–T6)	T4 (T3–T5)	0.190	
Bispectral index (20 min after i.v. dexmedetomidine)	76.3 (5.5)	75.4 (7.7)	0.629	0.9 (−2.9 to 4.7)

*Onset time (min)*				
To T10	5.3 (2.1)	5.6 (2.9)	0.699	−0.3 (−1.7 to 1.2)
To maximum sensory block	15.8 (5.3)	17.0 (6.5)	0.477	−1.2 (−4.6 to 2.2)
To Bromage 3 of motor block	10.0 (4.3)	10.2 (4.9)	0.879	−0.2 (−2.8 to 2.4)

*Recovery time (min)*				
To two-dermatome regression of sensory block	70.4 (10.2)	71.2 (12.4)	0.804	−0.8 (−7.2 to 5.6)
To four-dermatome regression of sensory block	97.2 (17.2)	98.8 (17.6)	0.747	−1.6 (−11.5 to 8.3)
To T10	102.8 (19.0)	106.4 (26.3)	0.582	−3.6 (−16.7 to 9.5)
To L1	130.4 (20.7)	131.2 (22.4)	0.896	−0.8 (−13.1 to 11.5)
To Bromage 2 of motor block	108.0 (25.7)	97.2 (24.8)	0.136	10.8 (−3.5 to 25.1)
To Bromage 1 of motor block	128.8 (26.7)	117.2 (26.2)	0.128	11.6 (−3.4 to 26.6)
Time to first micturition (min)	430.2 (121.5)	444.8 (95.0)	0.638	−14.6 (−76.6 to 47.4)

Data are presented as mean (SD) or median (IQR) of patients. *p* values are the results of unpaired *t*-test or Mann–Whitney *U* test between the groups.

**Table 3:** Intraoperative and postoperative side effects.

	Ropivacaine (*n* = 25)	Ropivacaine-fentanyl (*n* = 25)	*p* value
*Intraoperative*			
Hypotension	1 (4)	1 (4)	1.000
Bradycardia	14 (56)	15 (60)	1.000
Nausea	2 (8)	1 (4)	1.000
Vomiting	0 (0)	0 (0)	
Pruritus	0 (0)	1 (4)	1.000
Shivering	0 (0)	0 (0)	
Dyspnea	1 (4)	2 (8)	1.000

*Postoperative*			
Nausea	1 (4)	3 (12)	0.609
Vomiting	0 (0)	0 (0)	
Pruritus	1 (4)	0 (0)	1.000

Data are presented as mean (SD) or number (%) of patients. The *p* values are the results of the *χ*^2^ test or Fisher's exact test between the groups.

**Table 4:** Postoperative pain and requests of rescue analgesics.

	Ropivacaine (*n* = 25)	Ropivacaine-fentanyl (*n* = 25)	*p* value
Pain score at 1 hour after surgery	0 (0–0)	0 (0–0)	
Pain score at 24 hours after surgery	3.2 (1.8)	3.0 (1.7)	0.751
Number of patients requiring rescue analgesics within 24 hours after surgery	15 (60)	12 (48)	0.395
Time to first request of rescue analgesics (min)	176.8 (90.4)	252.0 (190.9)	0.081
Cumulative dose of the rescue analgesics for 24 hours after surgery, i.v. morphine equivalent dose (mg)	1.9 (1.9)	1.6 (2.1)	0.521
Cumulative dose of the analgesics for 24 hours including PCA, i.v. morphine equivalent dose (mg)	34.3 (4.0)	35.2 (4.4)	0.720

Data are presented as mean (SD), median (IQR), or number (%) of patients. The *p* values are the results of the Mann–Whitney *U* test for the continuous variables and *χ*^2^ test or Fisher's exact test for the incidence variables between the groups.

## Data Availability

It is being stored in the hospital data server. Due to personal information issues, it cannot be provided collectively. The authors will provide it separately if there is a later request.
